# Preferences for tongue swab-based versus sputum-based testing in the context of TB care: a best-worst scaling exercise in Vietnam and Zambia

**DOI:** 10.1136/bmjgh-2025-019092

**Published:** 2025-10-20

**Authors:** Maria del Mar Castro, Hien Le, Seke Muzazu, Nam Pham, Trang Trinh, Herbert Chabwera Nyirenda, Patricia Shabalu, Nora West, Ha Phan, Adithya Cattamanchi, Claudia M Denkinger, Monde Muyoyeta, Andrew D Kerkhoff

**Affiliations:** 1 Department of Infectious Disease and Tropical Medicine, Heidelberg University Hospital, Heidelberg, Germany; 2 German Center for Infection Research, Partner Site Heidelberg, Heidelberg, Germany; 3 Center for Promotion of Advancement of Society, Hanoi, Viet Nam; 4 Center for Infectious Disease Research in Zambia, Lusaka, Zambia; 5 Hanoi Lung Hospital, Hanoi, Viet Nam; 6 Division of Pulmonary Diseases and Critical Care Medicine, University of California San Francisco, San Francisco, California, USA; 7 Division of Pulmonary Diseases and Critical Care Medicine, University of California Irvine, Irvine, California, USA; 8 Division of HIV, Infectious Diseases, and Global Medicine, Department of Medicine, Zuckerberg San Francisco General Hospital and Trauma Center. University of California San Francisco, San Francisco, California, USA

**Keywords:** Tuberculosis, Diagnostics and tools, Global Health, Health Services Accessibility

## Abstract

**Background:**

The development of non-sputum-based tests is an urgent priority to increase access to tuberculosis (TB) diagnostic testing. Understanding preferences of people undergoing testing is critical for designing tests and strategies aligned with their needs.

**Methods:**

We conducted a survey and best-worst scaling (BWS) exercise to determine relative preferences for tongue swab-based versus sputum-based testing among people (≥13 years) with presumptive TB at primary health centres in Vietnam and Zambia. The BWS assessed 16 TB test features, including accuracy, sample type, turnaround time, cost and service aspects. We estimated mean rescaled preference weights, our primary outcome, using Hierarchical Bayes modelling and identified distinct preference groups using latent class multinomial logit analyses (LCA).

**Results:**

Among 409 participants enrolled, 356 (87%) met quality criteria for analysis. The median age of participants was 39 years (IQR 29–47), and most were female (60.7%). When asked directly, most participants preferred providing tongue swabs over sputum (58.1% vs 28.7%, p<0.001; 12.4% no preference). In the BWS exercise, tongue swab was also preferred over sputum (mean rescaled preference weights (MPWs) 6.4, 95% CI 5.9 to 6.8 vs 5.0 95% CI 4.6 to 5.4). However, support and counselling (MPW=14.0), sensitivity (MPW=12.3), specificity (MPW=10.2) and provider attitude (MPW=7.4) were the most important features overall. Less important features included facility opening hours (MPW=3.4) and the influence of trusted sources on testing decisions (MPW=2.2). LCA identified five distinct preference groups, but support and counselling were universally valued. Participants in Groups 2 (27.3%; n=97) and 3 (17.1%; n=61) valued tongue swabs over many other features. Group 5 participants (11%; n=39) strongly valued sputum-based testing.

**Conclusions:**

Participants in Vietnam and Zambia preferred tongue swab-based TB testing over sputum. However, sample type was less important than test accuracy and other TB care features affecting the testing experience.

**WHAT IS ALREADY KNOWN ON THIS TOPIC**

- Many novel tuberculosis (TB) assays using more readily accessible, non-sputum-based sample types show promising results. While the preferences of healthcare workers have been explored, those of people seeking TB care remain poorly understood, especially in high burden settings. Understanding their preferences for sample type and other features of the TB diagnostic process is crucial for informing people-centred TB care and expanding reach.

**WHAT THIS STUDY ADDS**

- This best-worst scaling experiment in Zambia and Vietnam shows that people prefer tongue swab tests over sputum samples. However, they placed much higher value on support and counselling, as well as test accuracy and provider attitude. These preferences were similar across both countries.

**HOW THIS STUDY MIGHT AFFECT RESEARCH, PRACTICE OR POLICY**

- The findings support the integration of tongue swab tests into TB diagnostic algorithms, while emphasising the need for comprehensive TB service delivery improvements. Policymakers can use these insights to develop more accessible and people-centred services aligned with the needs of those seeking TB care.

## Introduction

 Tuberculosis (TB) remains a global health challenge, contributing significantly to morbidity and mortality worldwide. The burden of TB is unevenly distributed, disproportionately affecting vulnerable populations such as individuals living in poverty, people living with HIV (PLHIV), migrants, refugees, people deprived of their liberty and children.^1^ These groups often face barriers to accessing timely and effective TB diagnosis and treatment, exacerbating health disparities and hindering global efforts to eliminate the disease.^2 3^

The development of non-sputum-based tests is an urgent priority to increase access to TB diagnostic testing. Conventional sputum-based methods are limited by their reliance on patients’ ability to produce sputum, which can be challenging for certain populations, including PLHIV and young children.^4^ In addition, sputum requires complex sample processing, which increases test costs and leads to longer turnaround times.^5^ The reliance on and limitations of sputum-based tests cause delays in diagnosis and treatment, which in turn contribute to ongoing transmission and poor health outcomes.^3 6 7^

Tongue swab sampling has emerged as a promising alternative for TB testing, particularly for people who are unable to produce sputum.^8^ Tongue swabs are easy to collect, including from PLHIV, people who are severely ill and children.^9 10^ Recent studies have demonstrated the feasibility of swab-based sampling for TB.^11^ Although diagnostic accuracy has varied and been less than that of sputum-based testing,^911^,^13^ a more accessible sample such as tongue swabs can yield a similar or higher number of people diagnosed with TB, as demonstrated in a recent meta-analysis of urine lipoarabinomannan testing in hospitalised people living with HIV.^5^

In addition to sample type, other features of the TB diagnosis process may be equally or more important to people affected by TB.^14^ Aspects related to quality of care, such as respect and communication from the providers, are important since negative healthcare experiences reported across low- and middle-income countries (LMICs) often stem from a lack of these elements and affect areas far beyond TB.^15^ The framework developed by the Lancet Global Health Commission to measure and improve the quality of TB care emphasises that expanding diagnosis and treatment coverage alone is insufficient to eradicate TB and that high-quality health systems are essential. Thus, values like people-centredness,^16^ equity, resilience and efficiency are critical to improve care^6^ and avoid excess deaths due to poor-quality services.^17^

Eliciting the preferences of people affected by TB through stated preference methods such as best-worst scaling (BWS) can provide valuable insights into the features that most strongly influence acceptance and widespread adoption of new diagnostic tools and approaches.^18 19^ Understanding people’s preferences can optimise TB test development efforts and improve TB care engagement and overall TB outcomes by making diagnostic processes more accessible and acceptable to those most in need.^19^ In this study, we aimed to estimate the relative preferences for tongue swab-based versus sputum-based molecular testing, and how they compared with other features related to TB service delivery and care in Vietnam and Zambia. These two WHO-designated high TB burden countries with distinct epidemiologic and health system profiles provided a valuable context to examine how preferences of TB-affected individuals vary across diverse healthcare settings.

## Methods

### Study design, setting and participants

We conducted a cross-sectional survey and BWS exercise assessing different TB test and care features in Vietnam and Zambia. Study sites were selected based on both scientific and pragmatic considerations. This evaluation was nested within a larger pragmatic study assessing the yield of tongue swab-based versus sputum-based molecular testing in high TB burden settings, which provided access to existing research infrastructure and established ethical and regulatory pathways. Both Vietnam and Zambia are WHO-classified high TB burden countries, with TB incidence rates of 182 and 283 per 100 000 population, respectively.^20 21^ Despite this shared high burden classification, the countries represent contrasting healthcare contexts: Vietnam has lower HIV co-infection prevalence and higher universal health coverage compared with Zambia. Although molecular testing is widely available in both countries (>95%), a greater proportion of TB cases in Zambia are diagnosed clinically.^20 21^ We enrolled consecutive people ≥13 years of age presenting to primary health centres in the urban area of Hanoi, Vietnam (n=3) and Lusaka, Zambia (n=3) who met local TB testing requirements (ie, a person with presumptive TB). We excluded people unwilling or unable to provide informed consent or cognitively unable to complete the BWS based on their performance in a handbook-guided warm-up task.

### Best-worst scaling design

The BWS assessed 16 TB test features, including aspects related to sample type, cost, accuracy, service delivery and care. These features were selected by reviewing existing literature and collaborative discussions with local investigators to maximise relevance and cultural appropriateness. Table 1 presents the list of features and the wording used to describe each feature in the BWS. Online supplemental file 1 provides additional details on the process of feature selection and refinement.

**Table 1:** List of features and text used for the BWS

Feature	Description shown in the BWS
Test sample collection	
Tongue swab	The test uses a tongue swab sample (you must stick out your tongue so that it can be gently swabbed for 15 s)
Sputum sample	The test uses a sputum sample (you must cough thick mucus from the back of your throat into a cup)
Test accuracy	
Sensitivity (false negative)	When you DO have TB, the chance is very low that the test gives you an incorrect negative result (the test is very good at detecting TB when you have TB)
Specificity (false positive)	When you DO NOT have TB, the chance is very low that you receive unnecessary TB treatment for 6 months because the test gives you an incorrect positive result
TB testing process and waiting time for results	
Additional tests	No additional samples or tests are needed after the first test to confirm TB diagnosis (you would not need to come back another day for further testing)
Rapid results (30 min)	The results of the TB test are available rapidly, on the spot (for example, within 30 min)
Same day results (5 hours)	The results of the TB test are available the same day (for example, within 5 hours)
Waiting time at facility	You spend very little time waiting at the facility to get tested for TB (for example, less than 30 minutes)
Cost and accessibility	
Free	The test does not cost you anything (it is free)
Provider attitude	The healthcare worker who helps you with TB testing is kind and respectful
Extended opening hours	Convenient, extended opening hours are available at the facility for TB testing (for example, early morning, nights or weekends)
Community location	Convenient community locations are available for TB testing (for example, sites near home or work)
Counselling and privacy	
Support and counselling	You are well counselled about what will happen while taking the test, and once the results are available, you receive counselling and support on their meaning and what will happen next
Privacy and stigma	You can get tested for TB privately, without being seen by people who know you (for example, friends, colleagues or family)
Trusted source	A trusted family member, friend or community leader recommends you get tested for TB
Results notification	You can choose how the test results are returned to you (for example, in person at the facility, SMS or phone call)

BWS, best-worst scaling; TB, tuberculosis.

The BWS was designed using Lighthouse Studio software V.9.15.0 (Sawtooth software, USA) and used a near-Balanced Incomplete Block Design (BIBD). In a BIBD configuration (v, b, r, k, λ), ‘v’ represents the number of items displayed across ‘b’ questions, each containing ‘k’ items. This set-up achieves approximate one-way level balance with each item appearing ‘r’ times, and two-way level balance as each item pairs with every other item ‘λ’ times.^22^ This design ensured that each participant completed 1 of 500 random versions of the survey, each containing 12 questions (ie, choice tasks) that presented four features. All participants saw each feature at least three times, allowing for robust individual-level preference weight estimates. A single prohibition was implemented such that the two time-related features—same-day and rapid results—were not featured in the same question since the directionality of preference could be assumed. To understand the absolute importance of the 16 features, we used the dual-response indirect anchoring method,^22^ where each choice task was followed by a question that asked participants to indicate the importance (‘all, some, none are important’) of the four displayed features (online supplemental figure 1). Following the BWS exercise, participants were asked two questions to assess the perceived difficulty of completing the BWS and making choices in each task. A participant information booklet was developed to provide standardised explanations to the study participants about each of the features. All informational material were delivered, and data were collected in the participants’ preferred languages (Vietnam: Vietnamese; Zambia: Nyanja, Bemba or English).

To optimise the understanding and relevance of the BWS, a pilot test was initially conducted at each study site (n=4 participants in Vietnam, n=6 in Zambia). Feedback from the pilot was captured using a structured form that included aspects like time, layout of the BWS and participant information handbook, clarity and difficulty of understanding, as well as open-ended feedback for improvement. This information was used to refine the BWS, handbook (online supplemental file 2) and improve the quality of the translations.

### Procedures

During site visits, and based on recommendations from on-site health workers, trained study staff approached all individuals presenting to the study health centres who met the local criteria for presumptive TB and were referred for sputum-based TB testing. Prior to consenting procedures for this substudy, all participants attempted sputum production and underwent healthcare worker-led tongue swab collection for TB testing. After informed consent for substudy participation, trained study staff provided standardised explanations of the 16 TB testing features using a participant information booklet. Study staff then administered a brief survey capturing demographics and information related to TB testing experience including ease of sample production, discomfort, satisfaction and direct preference for sputum versus tongue swab collection. The BWS was administered after the survey. All data was collected using the Sawtooth Software Offline Surveys app.

### Sample size and sampling

In line with simulation-based guidance for BWS designs and the formula proposed by Lipovetsky *et al*,^23^ a minimum of 133 participants per country was required.^23^ This calculation assumed 16 total features, where each participant is shown 4 features per task and 12 choice tasks, and a specified 95% confidence level with 0.14 margin of error. We increased the target sample size to 200 per country (n=400 total) to improve precision and support subgroup comparisons, within available budget and study timeline constraints. The sample size calculation is detailed in online supplemental file 1.

### Statistical analysis

Prior to analysis, BWS data were assessed at the participant level to ensure quality responses. Any participant meeting two of the following three criteria had their data excluded from the analysis: (1) an individual root likelihood (RLH) less than the 95% RLH cut-off generated from 500 random responses in Sawtooth (suggesting they likely answered at random without understanding or evaluating the options in each task)^22^; (2) a BWS completion time less than 40% of the median overall time for respondents in that country (suggesting they rushed through each question without careful consideration of the options in each task); (3) or a self-response indicating that the BWS exercise was somewhat or very difficult to complete (suggesting they may not have fully comprehended the exercise).

We summarised participants’ baseline characteristics and direct preference survey responses descriptively. We compared differences in proportions with Fisher’s exact test. We performed Hierarchical Bayesian (HB) modelling to estimate the individual-level probability rescaled preference weights for each participant and included country as a covariate. We estimated MPWs and their 95% CIs for each TB testing feature overall and by country. The rescaled MPWs, which sum to 100, indicate the relative importance of TB testing features and allow for direct comparison (eg, feature X is two times as important as feature Y) since they are on the same scale.

We used latent class multinomial logit analysis (LCA) to characterise preference heterogeneity by identifying segments of participants (groups) that cluster according to distinct preferences. For the final model, the number of distinct preference groups was selected based on optimising statistical fit (information criterion), interpretability, group size (ensuring meaningful and reliable groups) and maximum membership probability compared with other solutions.^24 25^ HB and latent class analysis were conducted using Lighthouse software V.9.15.0 (as part of Sawtooth software).

### Patient and public involvement

Patients (individuals seeking TB care) were involved solely as study participants and did not contribute to the study design, planning or interpretation of results. However, local health professionals, researchers and organisations were consulted in designing and carrying out study activities and interpreting results.

## Results

### Participant characteristics

Between September 2023 and February 2024, 423 eligible people were invited to participate in Vietnam and Zambia, of whom 409 completed the survey and BWS exercise. Based on the BWS quality criteria, 43 were excluded from Zambia and 10 from Vietnam, leaving 356 participants included in the final analysis (online supplemental figure 2). Excluded participants were younger, mainly from Zambia and more likely to report being unemployed (online supplemental table 1).

Overall, participants had a median age of 39 years (IQR 29–47) and the majority were female (60.7%, table 2). Most participants had at least some secondary-level education and were either self-employed (30.6%) or unemployed (21.1%); 45.5% of participants had previously been tested for TB, 14.3% had received prior treatment for TB, 25% self-reported having a positive HIV status and 2.2% self-reported having diabetes. When comparing characteristics across countries, people from Vietnam were more likely to be older, have higher education status, be employed, have been previously tested for TB and self-report being HIV-positive (table 2).

**Table 2:** Participant demographic and clinical characteristics

Characteristic	Overall	Vietnam	Zambia
	n=356	n=188	n=168
Age, years, median (IQR)	39 (29–47)	42 (37–49)	32 (25–43)
Female sex, n (%)	216 (60.7)	109 (58.0)	107 (63.7)
Education, n (%)			
Never attended school	8 (2.2)	0 (0.0)	8 (4.8)
Primary school	63 (17.7)	7 (3.7)	56 (33.3)
Secondary school	188 (52.8)	97 (51.6)	91 (54.2)
Higher level	97 (27.2)	84 (44.7)	13 (7.7)
Employment, n (%)			
Not employed	75 (21.1)	18 (9.6)	57 (33.9)
Yes, informal work	68 (19.1)	34 (18.1)	34 (20.2)
Yes, office job	58 (16.3)	41 (21.8)	17 (10.1)
Yes, self-employed	109 (30.6)	63 (33.5)	46 (27.4)
Other	27 (7.6)	26 (13.8)	1 (0.6)
Student	19 (5.3)	6 (3.2)	13 (7.7)
Prior testing for TB, n (%)			
Yes	162 (45.5)	93 (49.5)	69 (41.1)
No	189 (53.1)	90 (47.9)	99 (58.9)
Not sure	5 (1.4)	5 (2.7)	0 (0.0)
Prior TB treatment, n (%)			
Yes	51 (14.3)	30 (16.0)	21 (12.5)
No	305 (85.7)	158 (84.0)	147 (87.5)
HIV status*†, n (%)			
Positive	89 (25.0)	54 (28.7)	35 (20.8)
Negative	168 (47.2)	49 (26.1)	119 (70.8)
Not sure	98 (27.5)	85 (45.2)	13 (7.7)
Diabetes*, n (%)			
Yes	8 (2.3)	6 (3.2)	2 (1.2)
No	343 (96.3)	177 (94.1)	166 (98.8)
Not sure	5 (1.4)	5 (2.7)	0 (0.0)

* Self-reported.

† One participant declined to answer.

TB, tuberculosis.

### Sample collection and sample type preference survey

Nearly all participants were able to provide both sputum and a tongue swab sample; only 3 (0.8%) were unable to provide sputum, all from the Zambia site. When asked directly, most participants preferred providing tongue swabs over sputum (58.1% vs 28.7%, p<0.001), while 12.4% reported no preference. In both countries, a similar proportion of participants favoured tongue swab collection, while a higher proportion in Zambia preferred sputum compared with Vietnam (36.3% vs 21.8%, table 3). Compared with sputum, tongue swabs were reported as the easier sample to collect by 74.4% of participants and were associated with lower self-reported discomfort (10.1% vs 50.8%, p<0.001) and higher satisfaction (71.1% vs 49.2% very satisfied, p<0.001). These trends were consistent in both country-level analyses conducted in Vietnam and Zambia (table 3).

**Table 3:** Direct preferences between tongue swab and sputum testing, overall and per country

Variable	Overall	Vietnam	Zambia	P value
	n=356	n=188	n=168	
**Tongue swab sample**				
Discomfort or difficulty providing the sample, n (%)	36 (10.1)	8 (4.3)	28 (16.7)	<0.001
Overall satisfaction with providing the sample, n (%)				
Very satisfied	253 (71.1)	161 (85.6)	92 (54.8)	<0.001
Somewhat satisfied	88 (24.7)	25 (13.3)	63 (37.5)	
A little satisfied	14 (3.9)	1 (0.5)	13 (7.7)	
Not satisfied at all	1 (0.3)	1 (0.5)	0 (0.0)	
**Sputum sample**				
Discomfort or difficulty providing the sample, n (%)				
Yes	181 (50.8)	66 (35.1)	115 (68.5)	<0.001
No	172 (48.3)	122 (64.9)	50 (29.8)	
Not applicable	3 (0.8)	0 (0.0)	3 (1.8)	
Overall satisfaction with providing the sample, n (%)				
Very satisfied	175 (49.2)	84 (44.7)	91 (54.2)	0.06
Somewhat satisfied	109 (30.6)	68 (36.2)	41 (24.4)	
A little satisfied	60 (16.9)	32 (17.0)	28 (16.7)	
Not satisfied at all	9 (2.5)	4 (2.1)	5 (3.0)	
Not applicable	3 (0.8)	0 (0.0)	3 (1.8)	
**Preference regarding samples**				
Easier sample to provide, n (%)				
Sputum	37 (10.4)	19 (10.1)	18 (10.7)	0.32
Tongue swab	265 (74.4)	141 (75.0)	124 (73.8)	
No difference	51 (14.3)	28 (14.9)	23 (13.7)	
No data/not applicable	3 (0.8)	0 (0.0)	3 (1.8)	
Preferred sample to provide for TB diagnosis, n (%)				
Sputum	102 (28.7)	41 (21.8)	61 (36.3)	<0.001
Tongue swab	207 (58.1)	112 (59.6)	95 (56.5)	
No preference	44 (12.4)	35 (18.6)	9 (5.4)	
No data/not applicable	3 (0.8)	0 (0.0)	3 (1.8)	

TB, tuberculosis.

### Preferences for TB testing

**Figure 1:**
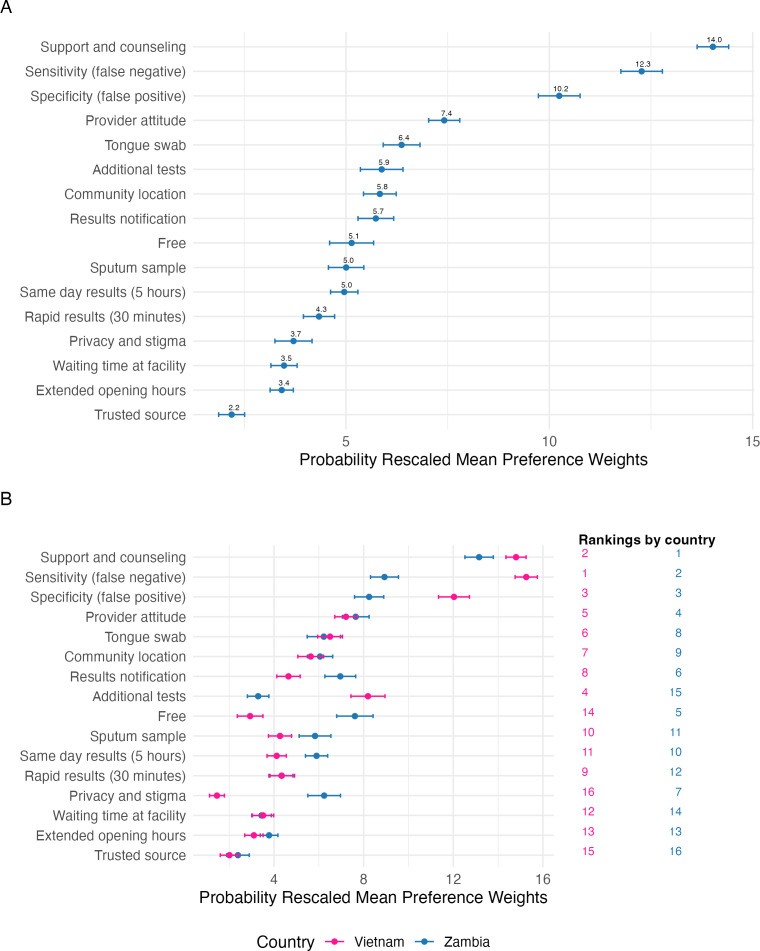
Probability rescaled average preference weights (n=356) for the overall cohort (**A**) and by country (**B**). Preference weights are rescaled to sum to 100. Differences should be interpreted descriptively, considering features with non-overlapping 95% CIs as meaningfully distinct. Small differences should be interpreted with caution.

All features assessed in this BWS were above the anchoring point, indicating that all were considered important by participants (online supplemental table 2). The most important features to participants were good support and counselling (MPW=14.0, 95% CI 13.6 to 14.4), high sensitivity (MPW=12.3, 95% CI 11.8 to 12.8), high specificity (MPW=10.2, 95% CI 9.7 to 10.8) and a kind and respectful provider (MPW=7.4, 95% CI 7.0 to 7.8) (figure 1A). Participants also valued not needing to undertake additional tests, a community testing location, free testing and a choice for how they receive testing results—each had an MPW between 5.0 and 5.9. Less important features were short waiting times (MPW=3.5, 95% CI 3.2 to 3.8), extended facility hours for testing (MPW=3.4, 95% CI 3.1 to 3.7) and the influence of trusted sources on testing decisions (MPW=2.2, 95% CI 1.9 to 2.5). In terms of sample type, tongue swab was preferred over sputum (MPW=6.4, 95% CI 5.9 to 6.8 vs 5.0 95% CI 4.6 to 5.4), with a relative rank of 5 versus 10 out of 16, respectively (figure 1A).

Country-specific estimates revealed that participants in Zambia had stronger preferences for free services (MPW=7.5, 95% CI 6.8 to 8.4 vs 3.0 95% CI 2.4 to 3.5), a choice of results notification (MPW=6.9, 95% CI 6.3 to 7.6 vs 4.6 95% CI 4.1 to 5.2) and enhanced privacy (MPW=6.2, 95% CI 5.5 to 7.0 vs 1.4 95% CI 1.1 to 1.8), while participants in Vietnam had stronger preferences for avoiding additional tests (MPW=8.3, 95% CI 7.4 to 9.0 vs 3.2 95% CI 2.8 to 3.8) (figure 1B).

Compared with older participants (30–49.9, and ≥50 years), younger participants (<30 years) placed a higher value on enhanced privacy, free services and extended facility hours, and a lower value on TB test accuracy (online supplemental figure 3). Participants who reported living with HIV had stronger preferences for test sensitivity (MPW=13.5, 95% CI 12.6 to 14.5 vs MPW=10.6 95% CI 9.8 to 11.4), specificity (MPW=11.4, 95% CI 10.5 to 12.5 vs MPW=9.3 95% CI 8.6 to 10.1) and avoiding additional tests (MPW=7.7, 95% CI 6.5 to 8.8 vs MPW=4.6 95% CI 4.0 to 5.3); those living with HIV also valued sputum samples less (MPW=3.8, 95% CI 3.0 to 4.7 vs MPW=5.6 95% CI 5.0 to 6.3) (online supplemental figure 4). No differences in MPW were observed by sex or prior TB treatment status (online supplemental figures 5 and 6).

### Heterogeneity of preferences (LCA)

**Figure 2:**
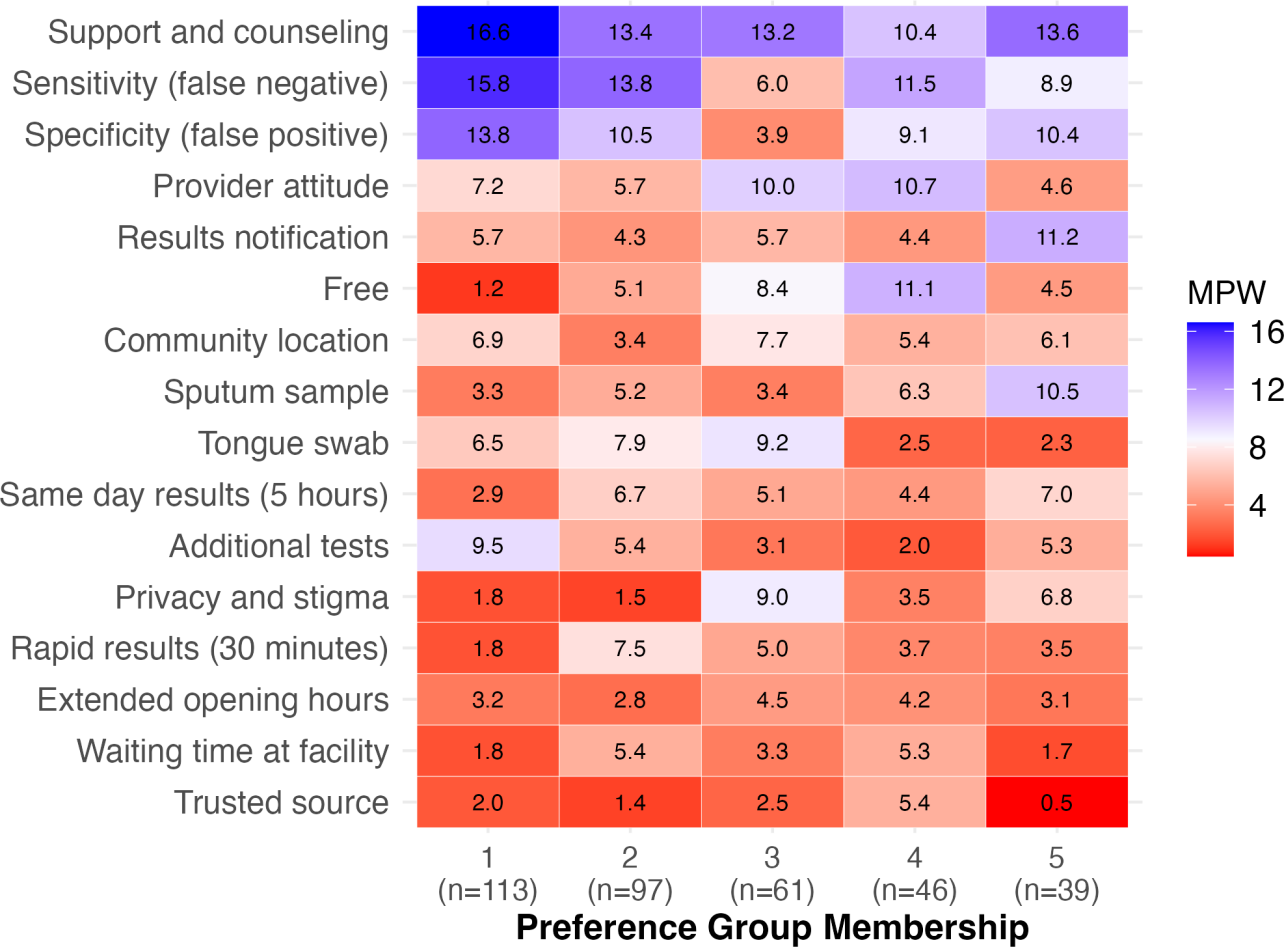
Mean preference weights for tuberculosis testing features according to latent class preference group. MPW, mean preference weights.

LCA identified five preference groups with distinct preferences regarding TB diagnostic service features (figure 2). Support and counselling were highly valued across all groups, while extended opening hours for facility-based testing and recommendations from a trusted source were consistently less valued features. Participants in Group 1 (‘Supportive, Single-Time, and Accurate’; n=113, 31.7%) valued support and counselling more than any other group and strongly valued high test accuracy, as well as not having to return for additional tests. Group 2 (‘Quick, Accurate, and Less-Invasive’; n=97, 27.3%) valued high test accuracy, tongue-swab-based tests and quick turnaround times, including same-day results. Participants in Group 3 (‘Service-oriented, Convenient, and Less-Invasive’; n=61, 17.1%) strongly valued tongue-swab-based testing and several service delivery features, including provider attitude, free services, enhanced privacy and community-based testing; this group notably differed from the other four by placing relatively low value on high test accuracy. Members of Group 4 (‘Free, Friendly, and Accurate’; n=46, 12.9%) placed a high value on free services and provider attitude, as well as high test accuracy; while those in Group 5 (‘Result choice, Sputum Preference, and Accurate’; n=39, 11%) valued having a choice of how to receive test results, sputum-based tests and test accuracy.

Participant characteristics by latent class are shown in online supplemental table 3. Persons from Vietnam were substantially more likely to belong to Groups 1 and 3, while participants in Zambia were more likely to belong to Groups 2, 4 and 5. Groups differed with respect to age, education, employment status and HIV status; however, no differences in group membership were observed by sex, prior TB testing or treatment status, or self-reported diabetes status.

## Discussion

Our study measured people’s preferences for sample type and other service features related to TB testing in two high-burden countries. Most participants preferred tongue swabs over sputum samples when asked directly. However, while tongue swab-based testing was a valued feature and preferred over sputum-based testing, the relative importance of sample type was lower compared with other TB care features such as counselling and support, test accuracy and provider attitude. Further, the LCA identified five distinct preference groups, all of whom placed high value on support and counselling. These data show that while the development and implementation of non-sputum-based tests, including tongue swabs, are aligned with the preferences of people affected by TB, a single approach may not meet the needs of all individuals and that novel, non-sputum-based diagnostic tests should be coupled with a broader focus on comprehensive TB care models that prioritise person-centred care, including robust support and counselling services and compassionate provider interactions.

Regarding sample type, while most (74.4%) participants found tongue swabs easier to provide and experienced lower discomfort and higher satisfaction with tongue swab collection, only 58.1% preferred swabs over sputum, indicating that preferences are influenced by other factors beyond ease of sample production. These include differences in the perceived trustworthiness of a non-sputum-based test, as has been reported for urine lipoarabinomannan (LAM), due to how people conceptualise the association between the aetiology of TB and the source of the sample.^26^ In the urine LAM study, some participants questioned the plausibility that TB, a disease primarily affecting the chest, could be detected in urine.^26^ Further, perceptions about accuracy and familiarity with sputum-based testing, which has been the standard sample for TB diagnosis for more than 100 years, may also influence preferences for sputum-based testing. In a recent qualitative study, perceived diagnostic accuracy and familiarity with the samples were critical in shaping sample preference.^27^ Other factors that shaped the acceptability of tongue swabs included ease of use, time to test result, diagnostic yield, hygiene, risk of TB transmission during sample collection, as well as trust in healthcare workers and the health system.^27^ These considerations may in part explain the differences in our results and a multicountry discrete choice experiment (DCE) where participants had a small preference for sputum-based testing, although this varied by country and may also be partly attributed to most patients’ lack of experience with tongue swab-based TB testing.^28^

Another important factor driving preferences was accuracy. A recent multicountry evaluation of MTB Ultima and MiniDock MTB demonstrated that tongue swabs achieved higher sensitivity compared with smear microscopy (77.9% vs 59.1% for MTB Ultima, and 85.7% vs 67.1% for MiniDock MTB), while maintaining specificities exceeding 98% across all sample types. Both platforms met or surpassed WHO target product profile benchmarks for sputum-based and non-sputum-based diagnostics.^29^ These platforms offer the flexibility to work with multiple specimen types, including tongue or sputum swabs, require minimal hands-on time and deliver results rapidly (<45 min for MTB Ultima; <30 min for MiniDock MTB).^29^,^31^ Clear communication of these accuracy metrics to both providers and people seeking care, combined with endorsement by regulators and WHO, will be critical for supporting widespread uptake.

In our study, sample type was of less relative importance than other TB testing and care delivery features. These findings align with a recent multicountry DCE, which showed that sample type was the least preferred feature of a TB test compared with other aspects like accuracy, cost, time to results and location of testing.^28^ Notably, the present study extends on this study by quantifying the value of features of the entire TB testing and care delivery experience rather than only the TB test itself. Supportive, respectful interactions with providers, including clear communication and counselling before and after testing, were highly valued in Vietnam and Zambia, and by all five latent class groups. This highlights the critical role of person-centred care in TB diagnostic services, which is highly aligned with the END TB strategy,^16^ and recent qualitative studies that show the critical role of trust that healthcare providers and facilities have on people’s preferences.^27 32^ Attention, respect and effective communication from providers have been shown to influence care-seeking and retention throughout the TB care cascade^33^,^35^ and extend to other health conditions across LMICs, where negative healthcare experiences are often reported.^15^ Respectful care, which encompasses valuing individuals’ privacy, confidentiality, dignity and demonstrating a compassionate attitude,^6^ aligns with our findings and emphasises the importance of person-centred approaches in facilitating care-seeking and treatment adherence.

Preferences for additional TB testing service features varied considerably within and between countries. These differences may reflect broader contextual factors such as health system structure and socioeconomic conditions. For example, stronger preferences in Zambia for free services and result notification options (in-person, vs SMS or phone call) correspond with higher poverty levels and lower health coverage.^20 21^ While HIV/TB co-infection is more prevalent nationally in Zambia, self-reported HIV status did not differ substantially between study populations. LCA revealed five distinct groups with differing priorities for features such as same-day results, having a choice of how results are received, enhanced privacy, free cost and not having to return for additional testing. While four of the five groups prioritised accuracy as a key feature, one group (Group 3, ‘Service-oriented, Convenient, and Less-Invasive’) accounting for 17% of participants placed a much lower emphasis on high test accuracy, instead valuing aspects such as tongue-swab-based testing, provider attitude, free services, enhanced privacy and community-based testing. This finding challenges the assumption that accuracy is universally the most prioritised feature and reflects a small subgroup potentially willing to trade lower accuracy for convenience of testing and service; it emphasises the need for novel tests that are convenient, affordable and easy to collect, even if they have lower accuracy. Preferences also differed to some extent by demographic factors. For instance, HIV-positive individuals prioritised accuracy and minimised additional testing, while young adults valued privacy, free services and extended hours. Collectively, this heterogeneity highlights the importance of tailoring TB diagnostic tests and services to better appeal to and effectively reach diverse populations, which is crucial for maximising TB diagnosis and promoting health equity.

Our study has several strengths. It was conducted in two diverse high-burden countries, enhancing the generalisability of the findings, particularly for commonly preferred and non-preferred features. Additionally, the use of standardised procedures in both countries ensured consistency and comparability, while BWS refinements through discussions with country partners and piloting contributed to local relevance and high comprehension. Furthermore, the study maintained high data quality, with less than 13% of data excluded due to low quality. Finally, having all participants experience both sample types helped minimise the hypothetical bias often found in choice experiments.^36^ However, the study also had some limitations. Study participants consisted exclusively of individuals accessing healthcare facilities, excluding individuals who did not seek care, could not access primary health centres or did not meet local TB testing requirements. This facility-based recruitment approach, while common in preference studies focused on patients or service users,^37^ differs from community-based studies that recruit from broader populations, including individuals not currently engaged with the health system who may have earlier and less symptomatic TB disease. Understanding the preferences of such persons is crucial as they represent those being missed by current TB services and should be prioritised in future research.^38^ They face different barriers^39^,^41^ and may benefit more from alternative sampling strategies since sputum collection in community settings may be challenging due to, among other factors, stigma, inability to produce and, issues handling the sample in field settings.^42 43^ Some subgroups, such as young adults and teenagers, were likely underpowered to detect differences. Also, the BWS used text descriptions without graphics, which may have led to some misunderstanding or misinterpretation despite extensive efforts by the team to explain the features and choice tasks during data collection; nevertheless, only 17% self-reported the BWS to be difficult. Although the selection of BWS features was informed by literature and local input, the final set was determined by the study team. While this may have introduced some bias, all features were consistently considered important by participants in the dual-response questions (with all MPW estimates exceeding the anchoring threshold), supporting their relevance and the validity of the selected features. Lastly, the self-reporting of HIV and diabetes status may have introduced minor biases, likely due to under-reporting, as individuals may not be aware of their HIV or diabetes status or may choose not to disclose it due to stigma.

In conclusion, participants undergoing TB testing in Vietnam and Zambia showed a clear preference for tongue swab-based testing over sputum-based testing. While tongue swab-based testing was a valued feature, the sample type was less important compared with other TB care features, including support and counselling, test accuracy and provider attitude, which were consistently more highly valued. The LCA revealed diverse preferences, including a group prioritising free costs and convenience over test accuracy, supporting the development of new tests that improve accessibility and population coverage even if they have lower accuracy. Our findings suggest that incorporating the preferences of people affected by TB into TB care models can better meet their needs and wants, ultimately improving diagnostic outcomes and satisfaction.

## Supplementary material

10.1136/bmjgh-2025-019092 online supplemental file 1

10.1136/bmjgh-2025-019092 online supplemental file 2

10.1136/bmjgh-2025-019092 online supplemental file 3

10.1136/bmjgh-2025-019092 online supplemental file 4

## Data Availability

Data are available in a public, open access repository.
