# Supplementary material for: Preferences for tongue swab-based versus sputum-based testing in the context of TB care: a best-worst scaling exercise in Vietnam and Zambia
Source: BMJ Glob Health. 2025 Oct 20;10(10):e019092. doi: 10.1136/bmjgh-2025-019092 (PMC12542534; doi:10.1136/bmjgh-2025-019092)
Supplement: online supplemental file 1 [file bmjgh-10-10-s001.docx]

# Supplementary File 1. Detailed methods

**Overview of BWS design**

This study employed a Best-Worst Scaling (BWS) Case 1 experiment to assess the relative importance of 16 predefined features. Each participant completed 12 choice tasks, with 4 features presented per task. The experimental design followed a near-Balanced Incomplete Block Design (BIBD) structure, ensuring that each feature appeared at least three times per participant and that feature co-occurrence was approximately balanced across tasks.

**Selection of features**

The BWS included 16 features reflecting aspects of TB test accuracy, sample collection, service delivery, accessibility, and person-centered care. An initial list of 19 candidate features was generated through reviewing existing literature and input from TB researchers and program implementers in Viet Nam and Zambia. These candidate features were refined through structured discussions with country teams and piloting at each study site to reduce redundancy, ensure clarity, and minimize participant burden.

For example, separate features on pre-test education and post-test support were merged into a single feature (support and counseling), while features such as inconclusive results, discomfort during sample collection, and recommendation by a family member or friend were excluded due to conceptual overlap or limited relevance for implementation. The final set of 16 features was selected based on policy relevance, local applicability, and feasibility of clear operationalization. A full list of candidate features and their final inclusion status is provided in Table 1.

# Table 1. Summary of candidate features and final inclusion status

| **Initial feature and wording** | **Final feature name** | **Status** | **Notes** |
| --- | --- | --- | --- |
| Tongue swab | Tongue swab | Included | Retained as-is |
| Sputum sample | Sputum sample | Included | Retained as-is |
| Sensitivity / False negative | Sensitivity (false negative) | Included | Retained as-is |
| Specificity / False positive | Specificity (false positive) | Included | Retained as-is |
| Inconclusive | — | Excluded | Conceptually overlapped with accuracy and less actionable, which makes it difficult to interpret in trade-offs |
| Additional test | Additional tests | Included | Retained as-is |
| Discomfort | — | Excluded | Overlapped with sample type and duplicative of non-BWS survey |
| Time to results – rapid | Rapid results (30 minutes) | Included | Retained with minor edits |
| Time to results – fast | Same-day results (5 hours) | Included | Retained with minor edits |
| Free | Free | Included | Retained as-is |
| Provider attitude | Provider attitude | Included | Retained as-is |
| Time at facility | Waiting time at facility | Included | Retained with minor edits |
| Hours | Extended opening hours | Included | Wording revised for clarity |
| Community location | Community location | Included | Retained as-is |
| Pre-test education | Support and counseling | Merged | These two features were merged due to conceptual similarities (both related to counselling and support) |
| Post-test support |  | Merged |  |
| Privacy/stigma | Privacy and stigma | Included | Retained with minor edits |
| Family/friend | Trusted source | Excluded | Wording revised for clarity |
| Results notification | Results notification | Included | Retained as-is |

**Sample Size Calculation**

The primary analytic objective was to estimate individual-level preference weights (utilities) using Hierarchical Bayesian (HB) modeling. Because standard frequentist sample size formulas are not directly applicable to HB estimation of utilities, we relied on the simulation-based and analytical framework described by Lipovetsky et al. (2015) to inform sample size requirements [1].

As a first step, we applied the Analytical Best-Worst (ABW) rule of thumb from Lipovetsky et al. (2015), which suggested a minimum of approximately 133 respondents per country would provide adequate precision for our design parameters. To formalize this estimate, we worked backward to determine the implied margin of error (δ ≈ 0.143) and confirmed this using their closed-form sample size formula.

**Formula for sample size calculation**

We used the following closed-form formula to confirm the minimum required sample size and margin of error [1]:


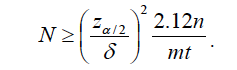


Where:

- N = minimum sample size required to achieve the specified margin of error for estimated preference weights
- Z_α/2_ = 1.96 for 95% confidence
- δ = is the margin of error (MOE)
- n = 16 is the total number of features
- m = 4 is the number of features per task
- t = 12 is the number of tasks per respondent
- 2.12 = simulation-derived constant (Lipovetsky et al. 2015)

Using a margin of error δ = 0.143, the minimum sample size required per country is:

N ≥ (1.96 / 0.143) ^2 * (2.12 × 16) / (4 × 12) ≈ 133

Thus, a sample size of 133 participants per country is sufficient to achieve an approximate MOE of 0.143, ensuring acceptable precision in the estimation of utility scores derived from the BWS design. Figure 1 presents the estimated margin of error for a range of sample sizes using the current design.


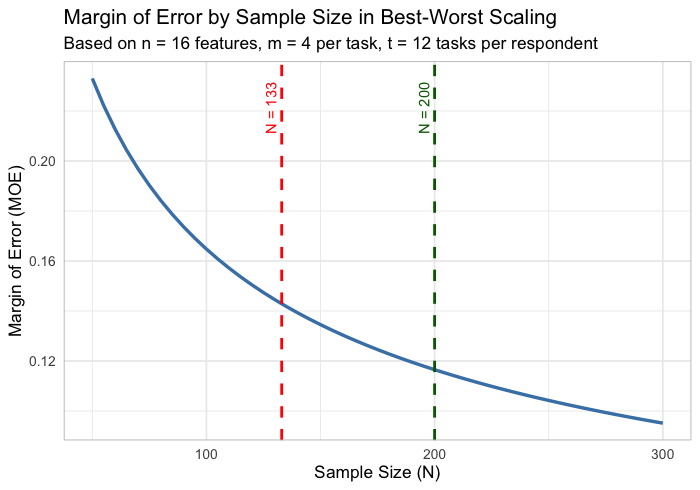


Figure 1. Margin of error by sample size estimations.

**Sample size rationale**

Although 133 participants per country would suffice for the intended precision, we increased the target to 200 per site (n=400 total) to enhance estimation accuracy (Figure 1), allow for subgroup analysis, and provide margin for data loss or nonresponse. This balanced statistical rigor with logistical feasibility.

**Assumptions and interpretive considerations**

*Assumptions:* While not powered for hypothesis testing between features, the sample size supports reliable estimation of relative utilities and enables meaningful interpretation of feature rankings in population-level and subgroup analyses. The assumptions listed below are relevant for analysis and interpretation:

- Feature mean preference weights (MPWs) are approximately normally distributed.
- Balanced exposure of features across participants (ensured by the near-BIBD design).
- Independence of responses across tasks within participants.
- HB estimation provides shrinkage-based individual utilities, which improves the stability compared to aggregate logit models.
- Rescaled MPWs are treated as interval-scaled and interpreted through observed differences and confidence intervals, rather than formal hypothesis testing.

*Stability in Rank Order*: Although not part of the formal sample size calculation, rank order stability was assessed descriptively by comparing the 95% confidence intervals (CIs) of the mean preference weights (MPWs). Features with non-overlapping CIs were considered meaningfully distinct and reliably ranked relative to one another. This approach is supported by prior simulation studies, which demonstrate that estimated ranks become more stable as sample size increases and standard errors are below 0.10 [1]. Rankings should be interpreted with caution when utility differences are smaller than the margin of error or when CIs overlap.

*Minimum Detectable Difference*: The study was not powered to detect statistically significant differences between features. However, differences between MPWs were interpreted descriptively: when their 95% confidence intervals did not overlap, features were considered meaningfully different. This criterion, while not a formal hypothesis test, supported the identification of features with higher relative preference, supporting practical prioritization.

**Data quality assessment and exclusion criteria**

Prior to analysis, BWS data were assessed at the participant level to ensure response quality. Any participant meeting two of the following three criteria was excluded: (1) a Root Likelihood (RLH) value below the 95% RLH threshold based on 500 random responses in Sawtooth (RLH= 0.350), indicating potential random or inattentive responding; (2) a completion time less than 40% of the country-specific median, suggesting insufficient engagement with the task; (3) self-reporting that the BWS tasks were somewhat or very difficult, indicating possible comprehension issues.

**Reference**

1. Lipovetsky S LD, Conklin M, editor What is the right size for my MaxDiff study? Sawtooth Software Conference Proceedings; 2015; Orlando, Florida. Orlando, FL, US: Sawtooth Software 2015.
