# Supplementary material for: Preferences for tongue swab-based versus sputum-based testing in the context of TB care: a best-worst scaling exercise in Vietnam and Zambia
Source: BMJ Glob Health. 2025 Oct 20;10(10):e019092. doi: 10.1136/bmjgh-2025-019092 (PMC12542534; doi:10.1136/bmjgh-2025-019092)
Supplement: online supplemental file 2 [file bmjgh-10-10-s002.pdf]

TSwaY BWS Participant Booklet

- As mentioned, we would like to ask you some questions to better understand your preferences when getting tested for TB. Various aspects, from the nature of the test itself to your experiences during the process, can influence your perception.

- This survey may be a bit different from others you may have done in the past. You will see several questions that each show 4 different features related to TB testing.

- For **each question**, we will ask you to tell us **which TB testing feature is the LEAST important** and **which is the MOST important to you**. There are no wrong answers as these questions are about what features of TB testing are most important to you.

- We hope to use this information in the future to design new TB tests and testing strategies that incorporate features people like yourself want and find the most acceptable.

First, I want to explain some of the different features of TB testing to help you better understand the survey.

Each question has three columns:

The middle column has **4** different features of the TB testing process we want you to think about and consider.

Least Important		Most Important
○	**Convenient community locations are available for TB testing** (for example, sites near home or work)	○
○	**You spend very little time waiting at the facility to get tested for TB** (for example, less than 30 minutes)	○
○	**The healthcare worker who helps you with TB testing is kind and respectful**	○
○	**The test uses a tongue swab sample** (you must stick our tongue so that it can be gently swabbed for 15 seconds)	○

the first column allows you to select **among these 4 features**, which one is **the LEAST important** to you

Least Important		Most Important
○	**Convenient community locations are available for TB testing** (for example, sites near home or work)	○
○	**You spend very little time waiting at the facility to get tested for TB** (for example, less than 30 minutes)	○
○	**The healthcare worker who helps you with TB testing is kind and respectful**	○
○	**The test uses a tongue swab sample** (you must stick our tongue so that it can be gently swabbed for 15 seconds)	○

The last column allows you to select **among these 4 features**, which one is **the MOST important** to you

You will see each option **appear more than one time in the survey**. This is normal. You will still need to consider between the available options because the combinations are always different.

Test sample type	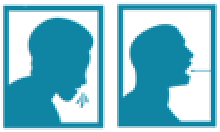
**The test uses a tongue swab sample**	For this test, a health worker will **gently rub your tongue** for 15 seconds with a soft cotton swab.
**The test uses a sputum sample**	For this test, you need to **cough into a cup** to collect thick mucus from the back of your throat

Test accuracy	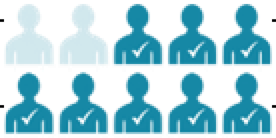
**When you DO have TB, the chance is very low that the test gives you an incorrect negative result**	A highly sensitive TB test means that **if you have TB disease, it's good at detecting TB** and is unlikely to miss it.
**When you DON'T have TB, the chance is very low that you receive unnecessary TB treatment for 6 months because the test gives you an incorrect positive result**	A highly specific TB test means that it's **good at confirming you DON'T have TB** when you really DON'T. You won't get treated for TB for 6 months if you don't need to be.

Time and efficiency of testing	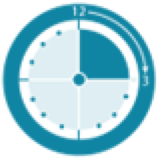
**No additional samples or tests are needed after the first test to confirm TB diagnosis**	**One test is enough** to find out if you have TB. You **won't need to worry about returning** to give more samples or take more tests.
**The results of the TB test are available rapidly, on the spot**	You will know the results of the TB test **within 30 minutes of providing a sample**.
**The results of the TB test are available the same day**	You'll get the results of your TB test **on the same day** you take it, but it may take up to 5 hours after you wait and provide a sample.
**You spend very little time waiting at the facility to get tested for TB**	**You'll be seen almost immediately**, within 30 minutes, allowing you to get on with your day.

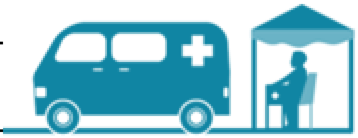

Affordability and convenience	
**The test does not cost you anything**	Taking the TB test is free so **you don't have to worry about paying** for it.
**The healthcare worker who helps you with TB testing is kind and respectful**	The person helping you with the TB test will **treat you with kindness and respect**. They will make sure you are comfortable.
**Convenient, extended opening hours are available at the facility for TB testing**	You can **pick a time that suits you**, even outside of regular business hours, like early mornings, nights, or weekends.
**Convenient community locations are available for TB testing**	You can get a TB test in the community **near where you live or work.** You don't need to go to the health facility.
**You can choose how the test results are returned to you**	You can **choose how to get your TB test results** - in person, by text, or by phone - whatever feels best for you.

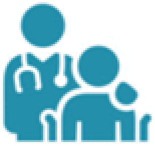

Privacy and support	
**You are well counseled about what will happen while taking the test, and once the results are available, you receive counseling and support on their meaning and what will happen next**	**Before and after the test, someone will explain everything and answer your questions** to help you understand what the results mean. You'll always know what to expect next.
**You can get tested for TB privately, without being seen by people who know you**	Your TB test will be done privately. **You won't have to worry about friends, family, or colleagues seeing you**.
**A trusted family member, friend, or community leader recommends you get tested for TB**	**Someone you trust and respect recommends getting a TB test**, which might make you feel more comfortable about it.
