# Supplementary material for: Preferences for tongue swab-based versus sputum-based testing in the context of TB care: a best-worst scaling exercise in Vietnam and Zambia
Source: BMJ Glob Health. 2025 Oct 20;10(10):e019092. doi: 10.1136/bmjgh-2025-019092 (PMC12542534; doi:10.1136/bmjgh-2025-019092)

Supplementary figures

Supplementary figure 1. Example of a BWS choice task

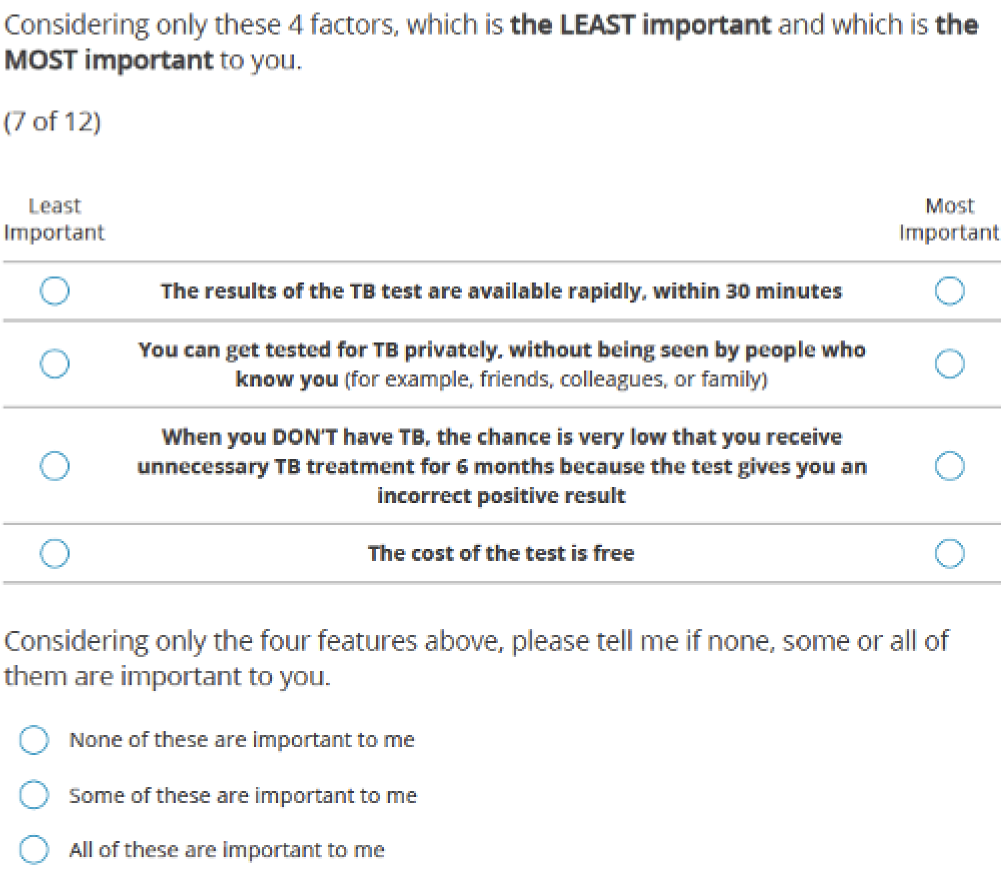

Considering only these 4 factors, which is **the LEAST important** and which is **the MOST important** to you.

(7 of 12)

Least Important		Most Important
◯	**The results of the TB test are available rapidly, within 30 minutes**	◯
◯	**You can get tested for TB privately, without being seen by people who know you** (for example, friends, colleagues, or family)	◯
◯	**When you DON'T have TB, the chance is very low that you receive unnecessary TB treatment for 6 months because the test gives you an incorrect positive result**	◯
◯	**The cost of the test is free**	◯

Considering only the four features above, please tell me if none, some or all of them are important to you.

- ◯ None of these are important to me
- ◯ Some of these are important to me
- ◯ All of these are important to me

Supplementary figure 2. Participant enrolment and inclusion

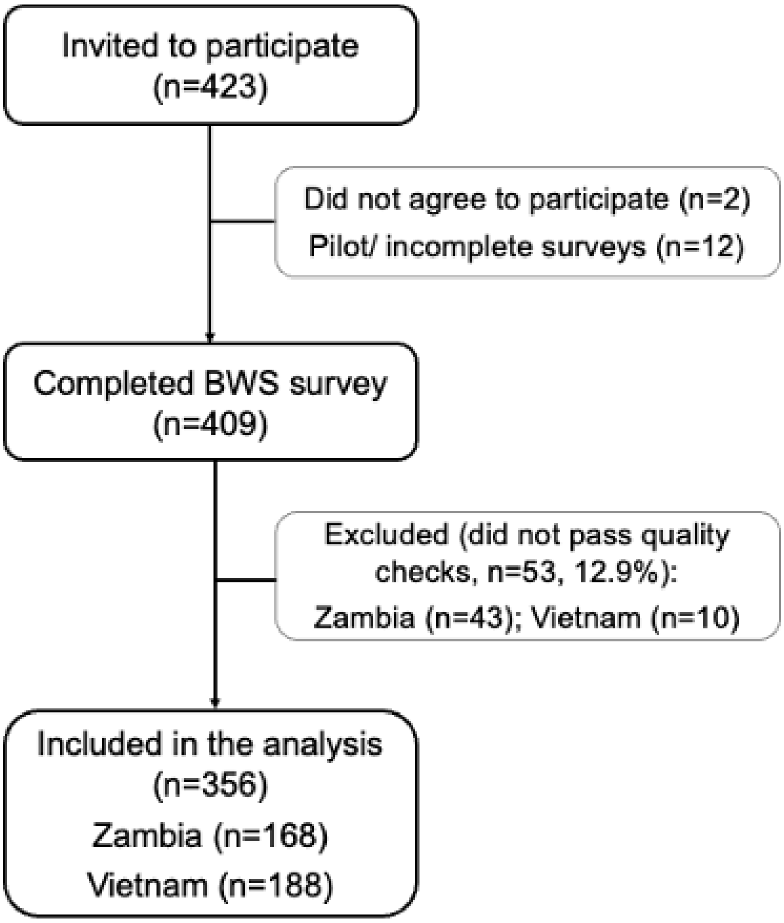

Supplementary figure 3. Mean preference weights by age group

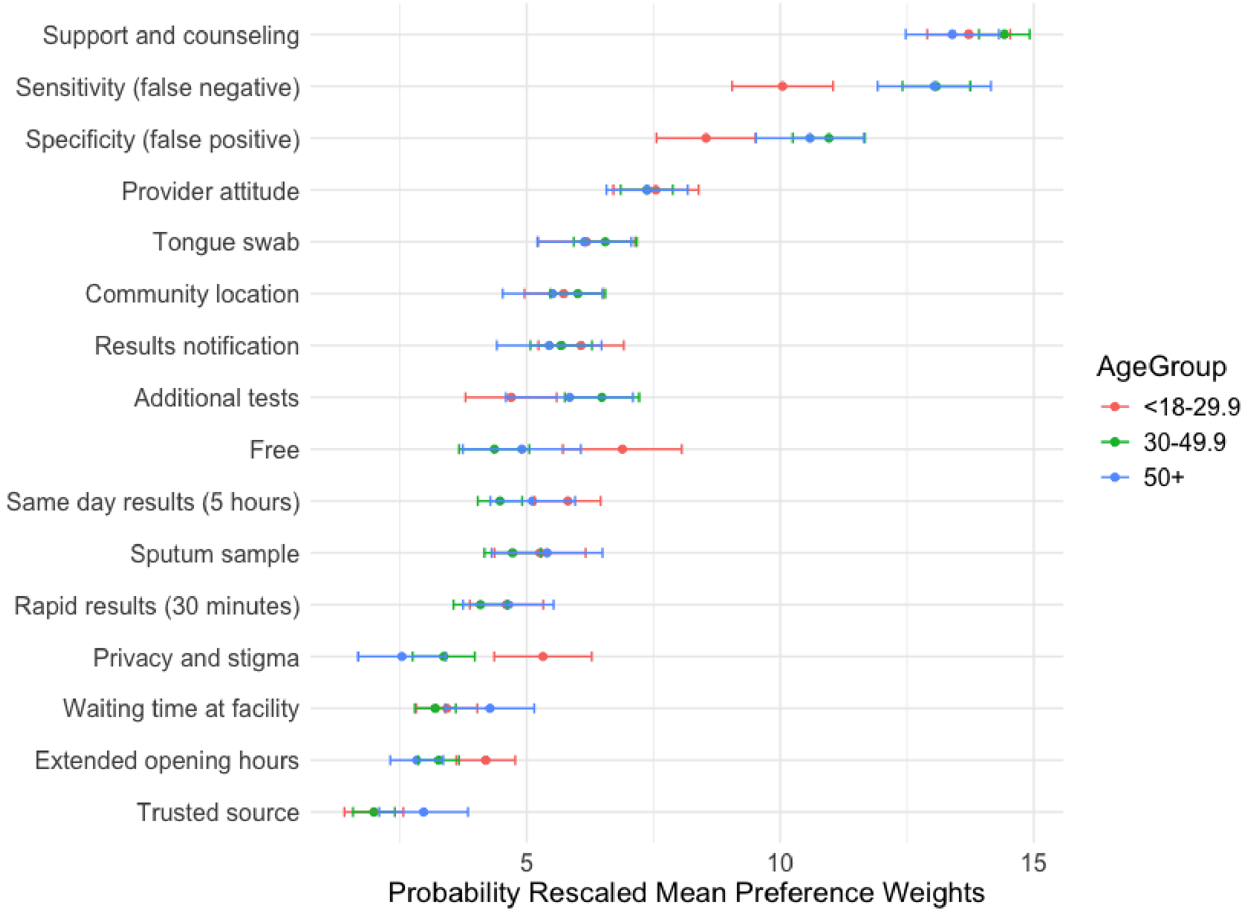

Supplementary figure 4. Mean preference weights by HIV status (n=257)

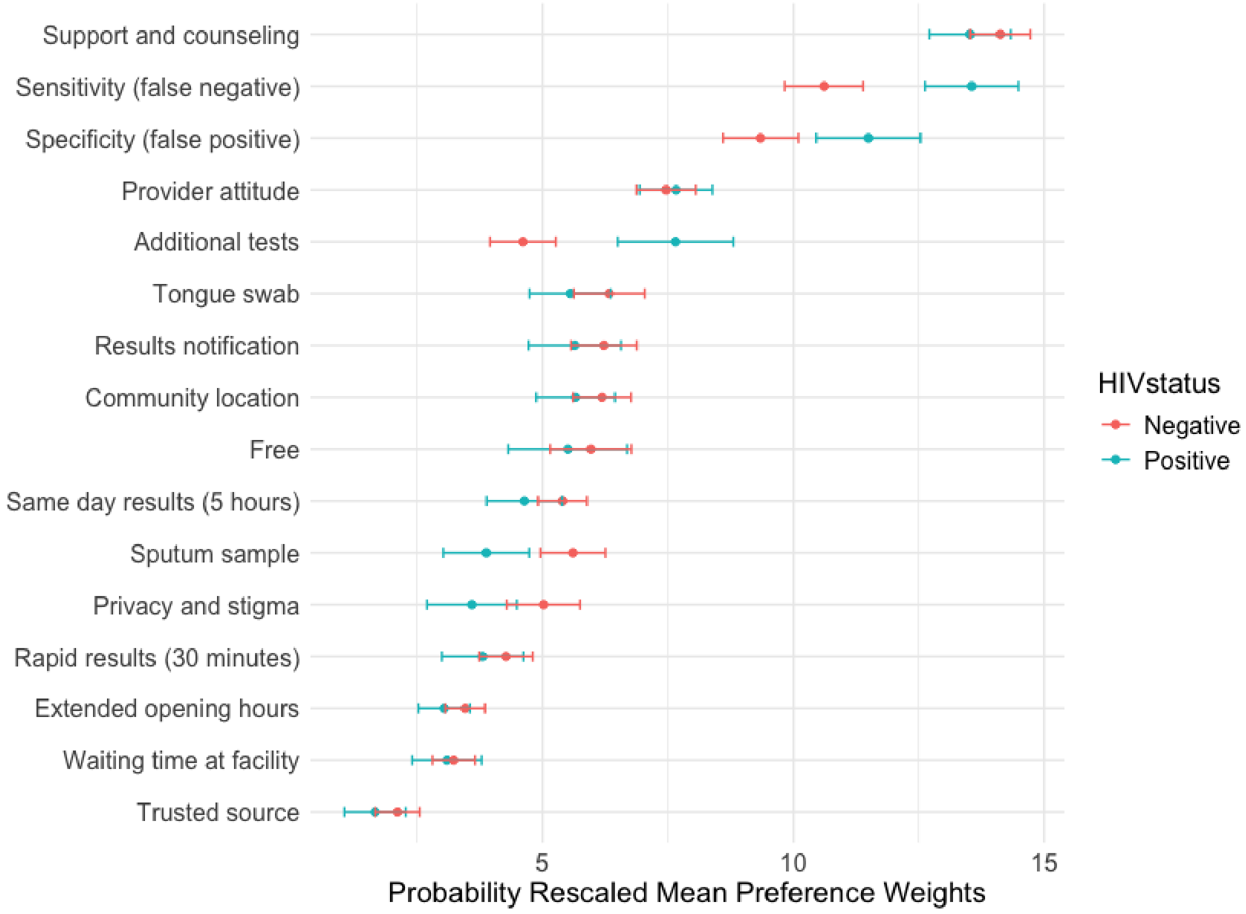

Supplementary figure 5. Mean preference weights by sex (n=356)

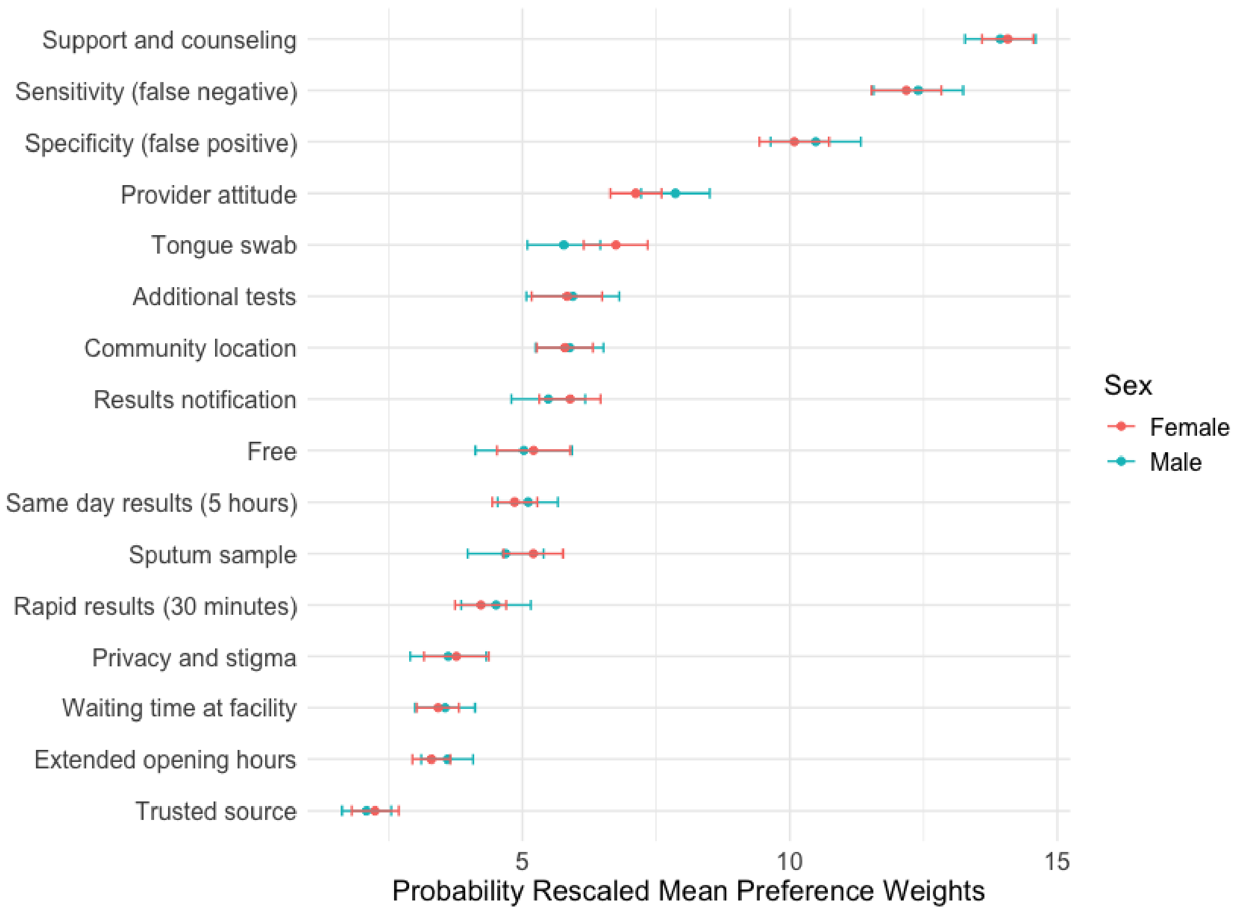

Supplementary figure 6. Mean preference weights by prior TB treatment status (n= 356)

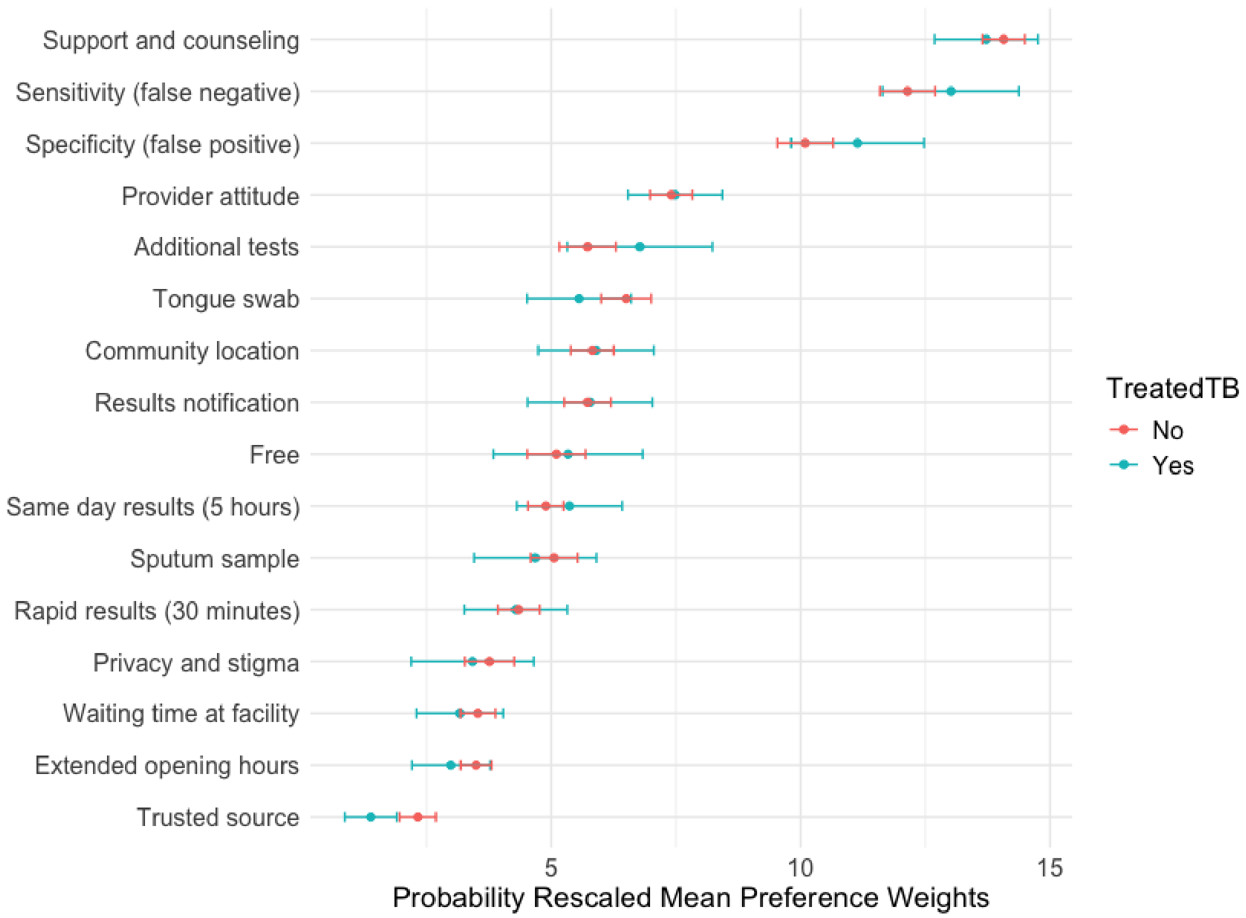

Supplement: online supplemental file 3 [file bmjgh-10-10-s003.pdf]
