# Supplementary material for: Preferences for tongue swab-based versus sputum-based testing in the context of TB care: a best-worst scaling exercise in Vietnam and Zambia
Source: BMJ Glob Health. 2025 Oct 20;10(10):e019092. doi: 10.1136/bmjgh-2025-019092 (PMC12542534; doi:10.1136/bmjgh-2025-019092)
Supplement: online supplemental file 4 [file bmjgh-10-10-s004.docx]

# **Supplementary tables**

**S1 Table. Baseline characteristics of study participants per inclusion/exclusion of the main analysis based on data quality**

| Characteristic | Included | Excluded | *p-value* |
| --- | --- | --- | --- |
|  | n=356 | n=53 |  |
| **Age,** years, Median (IQR) | 39 (28.5-47) | 31 (25-41) | 0.003 |
| **Female sex,** n (%) | 216 (60.7) | 32 (60.4) | 1 |
| **Country,** n (%) |  |  |  |
| Zambia | 168 (47.2) | 43 (81.1) | <0.001 |
| Vietnam | 188 (52.8) | 10 (18.9) |  |
| **Education,** n (%) |  |  |  |
| Never attended school | 8 (2.2) | 1 (1.9) | 0.09 |
| Primary school | 63 (17.7) | 16 (30.2) |  |
| Secondary school | 188 (52.8) | 28 (52.8) |  |
| Higher level | 97 (27.2) | 8 (15.1) |  |
| **Employment,** n (%) |  |  |  |
| Not employed | 75 (21.1) | 18 (34.0) | 0.02 |
| Yes, informal work | 68 (19.1) | 16 (30.2) |  |
| Yes, office job | 58 (16.3) | 4 (7.5) |  |
| Yes, self employed | 109 (30.6) | 13 (24.5) |  |
| Other | 27 (7.6) | 0 (0.0) |  |
| Student | 19 (5.3) | 2 (3.8) |  |
| **Prior testing for TB, n** (%) |  |  |  |
| Yes | 162 (45.5) | 19 (35.8) | 0.25 |
| No | 189 (53.1) | 34 (64.2) |  |
| Not sure | 5 (1.4) | 0 (0.0) |  |
| **Prior TB treatment**, n (%) |  |  |  |
| Yes | 51 (14.3) | 2 (3.8) | 0.06 |
| No |  |  |  |
| **HIV status *^#^,** n (%) |  |  |  |
| Positive | 89 (25.0) | 9 (17.0) | 0.004 |
| Negative | 168 (47.2) | 37 (69.8) |  |
| Not sure | 98 (27.5) | 6 (11.3) |  |
| **Diabetes***, n (%) |  |  |  |
| Yes | 8 (2.2) | 0 (0.0) | 0.37 |
| No | 343 (96.3) | 53 (100.0) |  |
| Not sure | 5 (1.4) | 0 (0.0) |  |

* Self-reported, # two people declined to answer

**S2 Table. Anchored mean preference weights using a Zero-Anchored Interval Scale (n=356)**

| **Feature** | **Mean** | **Lower 95% CI** | **Upper 95% CI** |
| --- | --- | --- | --- |
| Tongue swab | 48.83 | 45.57 | 52.09 |
| Sputum sample | 40.80 | 36.64 | 44.97 |
| Sensitivity (false negative) | 70.65 | 67.95 | 73.35 |
| Specificity (false positive) | 61.41 | 58.35 | 64.47 |
| Additional tests | 44.43 | 41.13 | 47.74 |
| Rapid results (30 minutes) | 40.86 | 37.16 | 44.56 |
| Same day results (5 hours) | 43.87 | 40.15 | 47.60 |
| Free | 39.27 | 34.50 | 44.04 |
| Provider attitude | 54.6 | 51.51 | 57.69 |
| Waiting time at facility | 36.01 | 32.07 | 39.95 |
| Extended opening hours | 36.98 | 33.37 | 40.58 |
| Community location | 45.97 | 42.38 | 49.57 |
| Support and counseling | 76.76 | 74.51 | 79.01 |
| Privacy and stigma | 32.62 | 28.19 | 37.06 |
| Trusted source | 25.73 | 21.95 | 29.51 |
| Results notification | 44.70 | 40.91 | 48.50 |
| **Anchor^+^** | **0.00** | **-** | **-** |

**^+^***Note: Anchored scores reflect absolute importance using a zero-anchored interval scale. The anchor (0.00) represents the threshold below which features are not considered important. All features evaluated scored above this threshold, indicating they were consistently considered important by participants in the dual-response questions.*

**S3 table. Baseline characteristics by LCA membership group**

| Variable | Preference Group Membership | | | | | *p-value* |
| --- | --- | --- | --- | --- | --- | --- |
|  | 1 | 2 | 3 | 4 | 5 |  |
| n= | 113 | 97 | 61 | 46 | 39 |  |
| **Country**, n (%) |  |  |  |  |  |  |
| Zambia | 19 (16.8) | 31 (32.0) | 49 (80.3) | 34 (73.9) | 35 (89.7) | <0.001 |
| Vietnam | 94 (83.2) | 66 (68.0) | 12 (19.7) | 12 (26.1) | 4 (10.3) |  |
| **Age**, Median (IQR) | 42 (33-48) | 41 (32-50) | 34 (25-40) | 39 (30-47) | 31 (25-46) | 0.002 |
| **Female sex,** n (%) | 66 (58.4) | 62 (63.9) | 37 (60.7) | 26 (56.5) | 25 (64.1) | 0.88 |
| **Education,** n (%) |  |  |  |  |  |  |
| Never attended school | 0 (0.0) | 0 (0.0) | 5 (8.2) | 2 (4.3) | 1 (2.6) | <0.001 |
| Primary school | 8 (7.1) | 10 (10.3) | 9 (14.8) | 18 (39.1) | 18 (46.2) |  |
| Secondary school | 65 (57.5) | 54 (55.7) | 34 (55.7) | 19 (41.3) | 16 (41.0) |  |
| Higher level | 40 (35.4) | 33 (34.0) | 13 (21.3) | 7 (15.2) | 4 (10.3) |  |
| **Employment**, n (%) |  |  |  |  |  |  |
| Not employed | 12 (10.6) | 24 (24.7) | 13 (21.3) | 18 (39.1) | 8 (20.5) | 0.002 |
| Yes, informal work | 19 (16.8) | 18 (18.6) | 11 (18.0) | 10 (21.7) | 10 (25.6) |  |
| Yes, office job | 20 (17.7) | 16 (16.5) | 9 (14.8) | 4 (8.7) | 9 (23.1) |  |
| Yes, self employed | 47 (41.6) | 19 (19.6) | 20 (32.8) | 12 (26.1) | 11 (28.2) |  |
| Other | 10 (8.8) | 14 (14.4) | 2 (3.3) | 1 (2.2) | 0 (0.0) |  |
| Student | 5 (4.4) | 6 (6.2) | 6 (9.8) | 1 (2.2) | 1 (2.6) |  |
| **Prior testing for TB,** n (%) |  |  |  |  |  |  |
| Yes | 57 (50.4) | 45 (46.4) | 23 (37.7) | 20 (43.5) | 17 (43.6) | 0.62 |
| No | 55 (48.7) | 49 (50.5) | 37 (60.7) | 26 (56.5) | 22 (56.4) |  |
| Not sure | 1 (0.9) | 3 (3.1) | 1 (1.6) | 0 (0.0) | 0 (0.0) |  |
| **Prior TB treatment**, n (%) |  |  |  |  |  |  |
| Yes | 19 (16.8) | 13 (13.4) | 8 (13.1) | 7 (15.2) | 4 (10.3) | 0.87 |
| No | 94 (83.2) | 84 (86.6) | 53 (86.9) | 39 (84.8) | 35 (89.7) |  |
| **HIV status*^#^**, n (%) |  |  |  |  |  |  |
| Positive | 33 (29.2) | 23 (23.7) | 14 (23.0) | 12 (26.1) | 7 (17.9) | <0.001 |
| Negative | 43 (38.1) | 34 (35.1) | 37 (60.7) | 23 (50.0) | 31 (79.5) |  |
| Not sure | 37 (32.7) | 40 (41.2) | 10 (16.4) | 10 (21.7) | 1 (2.6) |  |
| **Diabetes**, n (%) |  |  |  |  |  |  |
| Yes | 5 (4.4) | 1 (1.0) | 2 (3.3) | 0 (0.0) | 0 (0.0) | 0.53 |
| No | 106 (93.8) | 94 (96.9) | 58 (95.1) | 46 (100.0) | 39 (100.0) |  |
| Not sure | 2 (1.8) | 2 (2.1) | 1 (1.6) | 0 (0.0) | 0 (0.0) |  |
